# Conformational Stability of the N-Terminal Region of MDM2

**DOI:** 10.3390/molecules28227578

**Published:** 2023-11-14

**Authors:** Bruno Rizzuti, Olga Abian, Adrián Velazquez-Campoy, José L. Neira

**Affiliations:** 1 CNR-NANOTEC, SS Rende (CS), Department of Physics, University of Calabria, 87036 Rende, Italy; 2 Instituto de Biocomputación y Física de Sistemas Complejos (BIFI)—Unidad mixta GBsC-CSIC-BIFI, Universidad de Zaragoza, 50018 Zaragoza, Spain; oabifra@unizar.es (O.A.); adrianvc@unizar.es (A.V.-C.); 3 Instituto de Investigación Sanitaria Aragón (IIS Aragón), 50009 Zaragoza, Spain; 4 Centro de Investigación Biomédica en Red en el Área Temática de Enfermedades Hepáticas y Digestivas (CIBERehd), 28029 Madrid, Spain; 5 Departamento de Bioquímica y Biología Molecular y Celular, Universidad de Zaragoza, 50009 Zaragoza, Spain; 6 Instituto de Investigación, Desarrollo e Innovación en Biotecnología Sanitaria de Elche (IDIBE), Universidad Miguel Hernández, 03202 Elche, Spain

**Keywords:** MDM2, intrinsically disordered protein, conformational stability, circular dichroism, fluorescence, differential scanning calorimetry, constraint network analysis

## Abstract

MDM2 is an E3 ubiquitin ligase which is crucial for the degradation and inhibition of the key tumor-suppressor protein p53. In this work, we explored the stability and the conformational features of the N-terminal region of MDM2 (N-MDM2), through which it binds to the p53 protein as well as other protein partners. The isolated domain possessed a native-like conformational stability in a narrow pH range (7.0 to 10.0), as shown by intrinsic and 8-anilinonapthalene-1-sulfonic acid (ANS) fluorescence, far-UV circular dichroism (CD), and size exclusion chromatography (SEC). Guanidinium chloride (GdmCl) denaturation followed by intrinsic and ANS fluorescence, far-UV CD and SEC at physiological pH, and differential scanning calorimetry (DSC) and thermo-fluorescence experiments showed that (i) the conformational stability of isolated N-MDM2 was very low; and (ii) unfolding occurred through the presence of several intermediates. The presence of a hierarchy in the unfolding intermediates was also evidenced through DSC and by simulating the unfolding process with the help of computational techniques based on constraint network analysis (CNA). We propose that the low stability of this protein is related to its inherent flexibility and its ability to interact with several molecular partners through different routes.

## 1. Introduction

The 491-residue-long human version of MDM2 (mouse double minute 2, EC. 2.3.2.27) protein [1,2] is a negative regulator of p53, which is a tumor suppressor involved in apoptosis, DNA repair, senescence, and activation of genes implicated in cell cycle arrest [3,4]. MDM2 can interact with p53 through different ways: (i) the N-terminal region of MDM2 (N-MDM2), comprising the first 125 residues, interacts with the p53 N-terminal transactivation (TA) domain (residues 15–29), altering p53-transcriptional activity; (ii) the full-length MDM2 exports p53 from the nucleus with the help of an export signal in its sequence [2,5,6]; and (iii) the C-terminal region of MDM2 has a ubiquitin ligase function towards p53, activating p53 degradation via ubiquitin–proteasome pathways [3,7,8]. Thus, the MDM2/p53 interaction hampers p53 transcriptional regulatory activity and accelerates degradation of p53 within the proteasome [7]. On the other hand, there are various mechanisms that allow p53 to escape the interaction with MDM2 [9]. For instance, the protein ARF, whose expression is induced by the oncogenic proteins cMyc and Ras, is capable of binding to MDM2, abolishing its ability to bind p53 and hampering its p53-regulatory properties [10].

**Figure 1:**
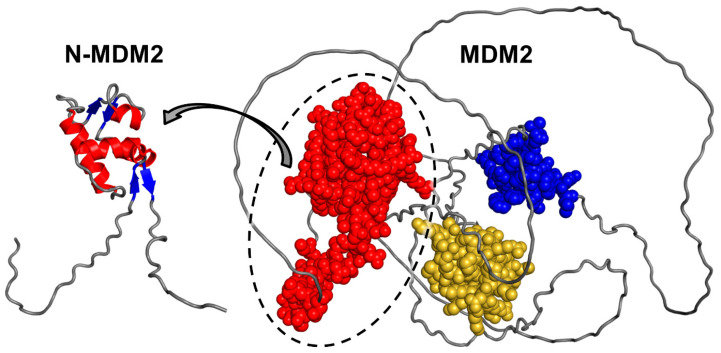
Structure of MDM2, as modeled by AlphaFold (entry Q00987). The three small well-folded domains are shown (sphere representation): N-terminal region N-MDM2 (red), including the p53 binding domain; zinc finger domain (blue); C-terminal RING domain (yellow). For N-MDM2 (circled), the details of the secondary structure are shown (inset on the left, having a different orientation), with α-helices in red and β-strands in blue.

From a structural point of view, MDM2 is an intrinsically disordered protein (IDP) which possesses long unfolded regions connecting three relatively small and well-folded domains (Figure 1). N-MDM2 includes the largest of them, which is the p53 binding domain (residues 25–109 [11]), and is composed by four α-helices and a smaller fraction of secondary structures organized in β-strands [6,12] (inset in Figure 1). The other two domains are a zinc finger (residues 300–330) and a C-terminal RING domain (starting from residue 438 onwards).

Studies aimed to understand the molecular mechanism behind the interplay between p53 and N-MDM2 have shown that the phosphorylation of residues in p53 is important in modulating such interaction and that the binding of different p53-derived ligands to N-MDM2 induces long-range conformational changes in the latter [13,14,15]. Furthermore, interactions with p53 occur through the movement of a so-called “lid”, which shifts in the presence of p53. Therefore, it seems that N-MDM2, in spite of being largely endowed with a structure, has an inherent and high conformational plasticity which could allow this protein to bind several different partners. In fact, N-MDM2 not only binds the TA of p53 but, among others, also the S100 proteins [16], the SAM domain of p73 [17], and the citrullinating enzyme PADI4 [18].

In this work, we studied the stability and conformational features of isolated N-MDM2 under different acidic pH conditions using several biophysical techniques, namely fluorescence, circular dichroism (CD), size exclusion chromatography (SEC), and differential scanning calorimetry (DSC). Furthermore, we modeled the simulated unfolding of N-MDM2 by using an analysis of the protein constraint network based on graph theory [19]. All the findings obtained with the different experimental techniques indicate that N-MDM2 has a low conformational stability and that it acquires a native-like conformation in a narrow pH range (between pH ~7.0 and 10.0). Despite its small size, thermal or chemical unfolding of N-MDM2 was found to be a complex process, showing the presence of several intermediates with different amounts of secondary and tertiary structures. The presence of these intermediates was also observed by the in silico experiments, which captured the occurrence of multiple sub-structures organized in a hierarchical fashion during the simulated denaturation process.

## 2. Results

### 2.1. N-MDM2 Acquires a Native-like Conformation in a Narrow pH Range

In order to measure the stability and describe the conformational features of N-MDM2, we ought first to determine in which pH range the domain acquired a native-like structure. To that end, we used several spectroscopic and biophysical probes, namely, intrinsic fluorescence, 8-anilinonapthalene-1-sulfonic acid (ANS) fluorescence, far-UV CD, and SEC. This whole set of techniques gives complementary information on different structural features of the polypeptide chain. In particular, we used intrinsic fluorescence to monitor changes in the tertiary structure of the protein around its eight tyrosine residues. We used ANS fluorescence to monitor the burial of solvent-exposed hydrophobic patches and to detect the presence of possible partially folded species [20]. We carried out far-UV CD experiments to monitor the changes in secondary structures. And, finally, we used SEC to determine the compactness of the polypeptide chain.

#### 2.1.1. Steady-State Fluorescence and Thermal Denaturations

**Figure 2:**
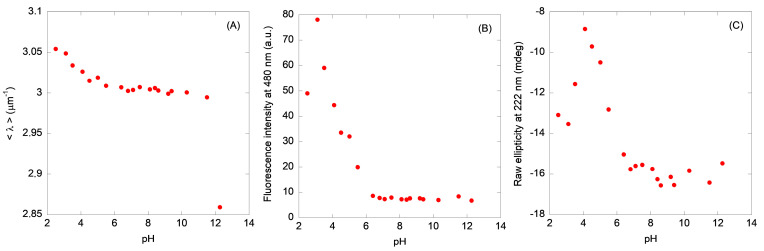
pH-induced structural changes in N-MDM2 followed by spectroscopic techniques: (**A**) variations in the intrinsic fluorescence of N-MDM2 monitored by the changes in the <λ>. (**B**) changes in intensity of the ANS binding followed at 480 nm for N-MDM2. The ANS concentration was 100 µM. (**C**) changes in molar ellipticity at 222 nm, from far-UV spectra of N-MDM2. Spectra were acquired in either 1 cm pathlength (fluorescence) or 0.1 cm pathlength cells (CD); buffer concentration was 10 mM in all cases. The curves represent the variation in a spectroscopic parameter (raw ellipticity at 222 nm for CD, and the <λ> or the intensity at a particular wavelength for fluorescence) vs. the pH.

The fluorescence spectra of N-MDM2 showed a maximum at 308 nm due to the presence of the eight tyrosine residues. The averaged emission intensity, <λ>, (Figure 2A) and the fluorescence intensity at 308 nm (Figure S1A) of N-MDM2 showed two transitions. The first occurred with a p*K*_a_ = 4.0 ± 0.3, obtained from the <λ> and the fluorescence intensity. The second one took place at basic pH values (pH > 10.0) probably due to the titration of tyrosines; in this transition, no basic baseline was observed and we could not determine the p*K*_a_. Then, N-MDM2 seemed to acquire a native-like conformation between pH 6.0 and 10.0.

Thermal denaturations performed at several pH values (pH = 4.1, 6.4, 8.6, and 11.5) were carried out by following the changes in the intrinsic fluorescence for N-MDM2. At any of these pH values, we could not detect any sigmoidal transition, and scattering of the fluorescence signal was observed at high temperatures (Figure S2).

#### 2.1.2. ANS-Binding Fluorescence

At low pH, the ANS fluorescence intensity at 480 nm was larger at acidic pH values and decreased as the pH was raised (Figure 2B), suggesting that under acidic conditions the domain had solvent-exposed hydrophobic regions. The intensity at 480 nm showed a sigmoidal-like behavior at pH > 3.0, yielding a p*K*_a_ of 4.7 ± 0.5. At pH < 3.0, another transition was observed, but we could not determine its midpoint due to the lack of an acidic baseline. The <λ> (Figure S1B) showed two transitions as well at acidic pH values; however, we could not determine the p*K*_a_ for the most acidic one due to the absence of a baseline. The other transition occurred at a p*K*_a_ of 5.8 ± 0.6, obtained from the experimental values of the spectral intensities measured between pH 4.5 and 8.0. Conversely to what happened for the intrinsic fluorescence, we did not observe any titration at basic pH values (by using either the fluorescence intensity or <λ>), indicating that the titration of tyrosines did not lead to the development of a large amount of nearby solvent-exposed hydrophobic patches.

#### 2.1.3. Circular Dichroism

The CD spectrum of N-MDM2 at pH 7.0 (Figure S3) showed a minimum at ~205 nm and another one with a smaller intensity at 222 nm, suggesting the presence of α-helix and β-sheet conformations, as also shown by the NMR structure of the native domain [12]. The spectrum at pH 4.5 was similar to that at pH 7.0, but with a smaller intensity. In general, the spectra had a lower intensity at pH < 6.0, indicating that at acidic pH values the domain had lost some of its secondary structure, resulting in a large amount of nearby solvent-exposed hydrophobic residues (as concluded from the experiments in the presence of ANS, Figure 2B). The changes in ellipticity at 222 nm (Figure 2C) were similar to those obtained by monitoring ANS fluorescence, showing the presence of two transitions. We could not determine the p*K*_a_ of the most acidic one, whereas the p*K*_a_ of the other was of 5.0 ± 0.8 (obtained from the experimental values of the ellipticity at 222 nm measured between pH 4.0 and 7.1), similar to that obtained from ANS measurements. Then, from the behavior of the ellipticity at 222 nm in the far-UV CD spectrum, we can conclude that the domain acquired a native-like conformation in the range between pH 7.0 and 9.0.

We also carried out thermal denaturations at the same pH values used for the fluorescence experiments (pH = 4.1, 6.4, 8.6, and 11.5). We only observed a complete (although irreversible) sigmoidal titration at pH 8.6, with a thermal denaturation midpoint of ~66 °C (Figure S4).

#### 2.1.4. SEC Experiments

**Figure 3:**
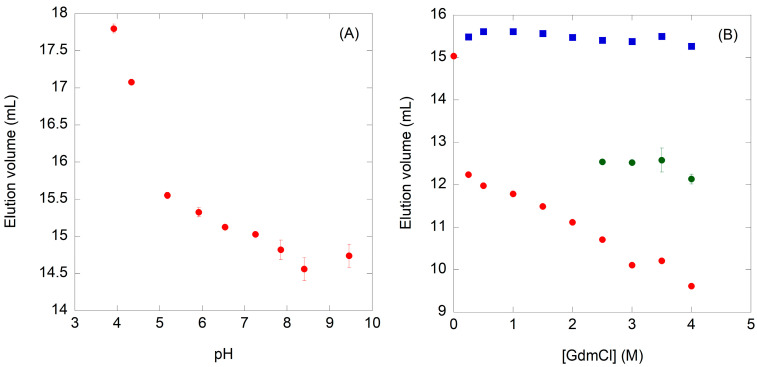
Structural changes in N-MDM2 induced by pH and GdmCl denaturation, followed by hydrodynamic methods (SEC). (**A**) changes in the elution volume (mL) of N-MDM2 as the pH was varied. The bed volume of the column was 18.98 mL, and at pH values < 4.0, the proteins eluted at volumes larger than the bed volume; these experimental elution volumes have not been represented. (**B**) changes in the elution volume of the domain as the GdmCl concentration was modified. The points correspond to elution volume of the protein species appearing at any concentration (red circles), at [GdmCl] ≥ 0.25 M (blue squares), and at [GdmCl] ≥ 2.5 M (green squares). The bars are the standard deviations from three different measurements for each pH or GdmCl concentration.

In SEC experiments performed at pH < 4.0, the protein did elute from the column at volumes (*V*_e_) larger than the bed volume (18.98 mL) (Figure 3A). This result suggests that N-MDM2, even though NaCl was present, was bound to the column at those acidic pH values. We know from the experiments with ANS and far-UV CD that at low pH values N-MDM2 had lost some of its secondary structure, resulting in nearby solvent-exposed hydrophobic patches (Section 2.1.2 and Section 2.1.3). We hypothesize that those partially unfolded, solvent-exposed, and highly hydrophobic regions can interact with the column, resulting in larger *V*_e_ values. In the pH range 4.0‒7.0, the *V*_e_ decreased until a constant volume was reached (15.06 ± 0.07 mL). Therefore, the protein did not attain a native-like compactness until a physiological pH was attained.

To sum up, all the biophysical characterizations suggest that: (i) N-MDM2 did not reach a native-like structure unless in proximity of pH ~7.0; and (ii) this native-like structure changed upon the titration of the tyrosine residues at pH > 10.0.

### 2.2. Conformational Stability of N-MDM2 at Physiological pH

We tried to measure the conformational stability of N-MDM2 at physiological pH (where it had acquired a native-like conformation at pH 7.0) by using DSC and chemical-denaturations monitored by fluorescence (both intrinsic and ANS fluorescence) and far-UV CD.

#### 2.2.1. Calorimetry and Thermal Denaturations with a Fluorescent Extrinsic Probe

**Figure 4:**
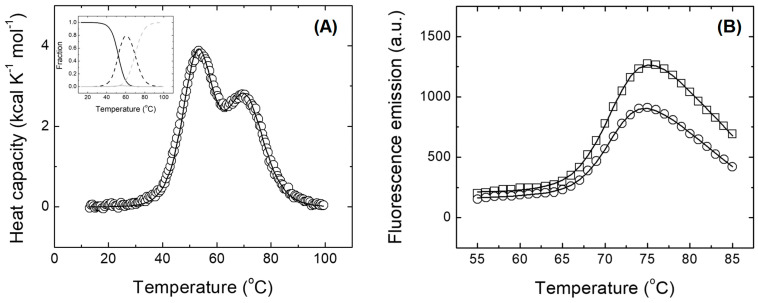
Differential scanning calorimetry and thermal denaturations followed by an external probe. (**A**) differential scanning calorimetry. The thermogram corresponding to the thermal unfolding of N-MDM2 was analyzed with a two-transition model. More complex models performed worse and did not converge. (Inset) Using the estimated thermodynamic parameters for each transition (*T*_m,i_, Δ*H*_i_(*T*_m,i_), and Δ*C*_P,i_), the populations of the different conformational states were calculated: native state (continuous black line), first intermediate state (dashed black line), second intermediate state (continuous gray line), and unfolded state (dashed gray line). (**B**) thermal denaturations followed by the fluorescence of the SYPRO Orange. Two unfolding traces are shown, corresponding to two different concentrations: 14 μM (circles) and 25 μM (squares). The analysis was performed employing the two-state (single-transition) model to estimate *T*_m_, Δ*H*(*T*_m_), and Δ*C*_P_.

Since all the biophysical probes agree with the fact that N-MDM2 acquired a native-like conformation between pH 7.0 and 10.0, we carried out DSC experiments at physiological pH (Figure 4A and Figure S5). Two transitions were observed, indicating the presence of two intermediates during calorimetric unfolding; the first transition had a *T*_m_ of 52.3 °C and an enthalpy value of 55 kcal mol^−1^; and the second one had a *T*_m_ of 69.8 °C and an enthalpy value of 50 kcal mol^−1^. Errors for the T_m_ are 0.2 °C, and those for enthalpies are 3 kcal mol^−1^. Regarding the unfolding heat capacity change and its associated error, we removed the step of the transition with a quasi-sigmoidal baseline to avoid any bias associated with that. In that way, the heat capacity change Δ*C*_P_ appears in the fitting equations but not explicitly as a geometric feature in the thermogram. The latter value of *T*_m_ was similar to that obtained from the far-UV CD data (above and Figure S4). We did not observe a concentration dependence during the experiment (that is, native and unfolded states of the protein were both monomeric). Furthermore, we did not observe any scanning-rate dependence in the scans (that is, there was no coupling between the kinetics of the temperature scanning and that of the protein unfolding reaction and, therefore, the process connected true equilibrium states). Based on the data obtained, the population of the first intermediate state in the unfolding almost reached 80% around 60 °C, but the population of the second intermediate was never larger than 2%.

On the other hand, the thermal unfolding of N-MDM2 monitored by fluorescence emission of an extrinsic fluorophore (SYPRO Orange) (Figure 4B) yielded a *T*_m_ of 71.3 °C, with an enthalpy value of 120 kcal mol^−1^. That single *T*_m_ value agrees with the second *T*_m_ detected by DSC and far-UV CD, and the enthalpy change is equivalent to the total unfolding enthalpy observed by DSC. Again, we did not observe a concentration dependence during the experiments (that is, native and unfolded states were monomeric).

These results suggest that the temperature-driven unfolding of N-MDM2 is complex in spite of the small size of this protein domain and has a conformational landscape containing at least two partially folded intermediates.

#### 2.2.2. Chemical Denaturation by Using Guanidinium Hydrochloride as Denaturant 

**Figure 5:**
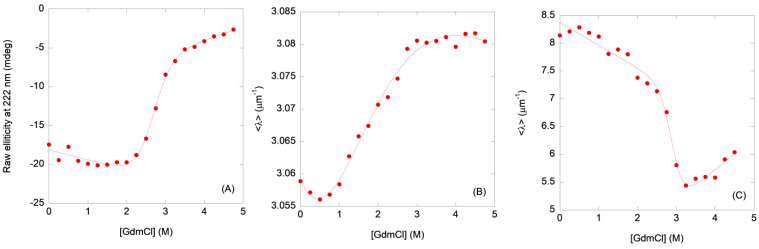
GdmCl-denaturation of N-MDM2 followed by spectroscopic techniques. The curves represent the variation in a spectrocopic parameter (raw ellipticity at 222 nm for CD and the <λ> for fluorescence) vs. the concentration of denaturant. (**A**) changes in the ellipticity at 222 nm (far-UV CD). (**B**) changes in the intrinsic fluorescence monitored by <λ>, after excitation at 280 nm. (**C**) changes in ANS fluorescence monitored by <λ>, after excitation at 370 nm.

To probe the chemical denaturation of N-MDM2, in a first attempt we tried to use urea as a chemical denaturant. Unfortunately, either followed by CD or intrinsic fluorescence (Figure S6), this resulted in a gradual, step-wise variation in both biophysical properties and no sigmoidal transition was observed. Therefore, we decided to use GdmCl as a chemical denaturant. The ellipticity at 222 nm (Figure 5A) and the <λ> of the intrinsic fluorescence (Figure 5B) yielded sigmoidal curves with different apparent [GdmCl]_1/2_ and *m*-values. The GdmCl denaturation followed by the fluorescence of ANS also showed sigmoidal behavior by monitoring intensity at 509 nm (Figure 5C): the apparent thermodynamic parameters calculated in the process were similar to those obtained from far-UV CD data (Figure 5A). Finally, attempts to follow the GdmCl-denaturation process by SEC yielded two peaks at concentrations > 0.25 M of denaturant (Figure 3B) and three peaks at GdmCl concentrations > 2.5 M, indicating the presence of different species which are separated by the column matrix.

### 2.3. Simulated Thermal Unfolding of N-MDM2

The unfolding process was modeled by using the constraint network analysis (CNA) algorithm [21], with the single-network topology generated by providing as input the N-MDM2 structure (residues 6–125) reported by AlphaFold [22]. In brief, CNA predicts the features of protein thermal unfolding by investigating the mechanical flexibility/rigidity of the macromolecule through modeling based on graph theory [19]. The thermal unfolding is simulated by focusing on the network of non-bonded interactions that defines the protein native state and gradually removing them to mimic a loss of structural rigidity. This removal proceeds at increasing energy E (having a positive sign in our case, as explained in Section 4). In this way, conformational transitions of the protein during the unfolding can be identified as abrupt rigid-to-flexible changes in the connectivity network. A distinctive feature of CNA is that it can capture the (possible) presence of multiple phase transitions along the unfolding pathway, especially for proteins with a more sensitive structure or complex architecture [21].

#### 2.3.1. Global Indexes in the Simulated Unfolding Process

**Figure 6:**
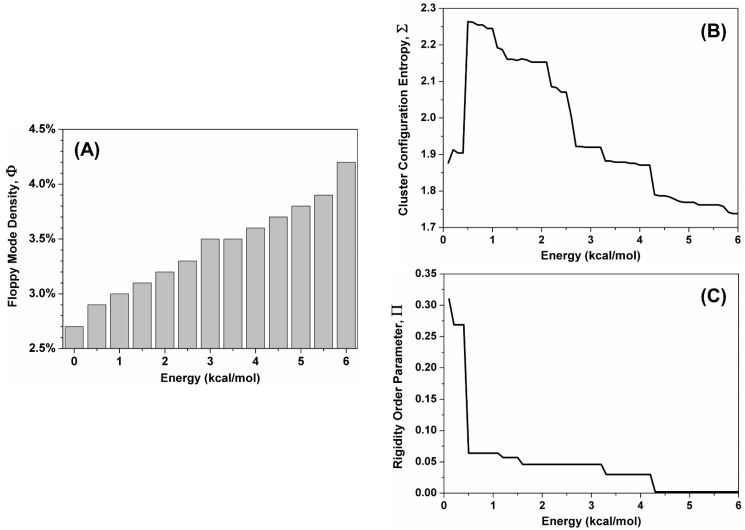
Global flexibility indexes of N-MDM2, as a function of the reaction coordinate of the unfolding process. (**A**) normalized number of inner degrees of freedom in the protein, Φ, also known as the floppy mode density. (**B**) cluster configuration entropy, Σ, describing the disorder of the network. (**C**) rigidity order parameter, Π, indicating the number of constraints in the network.

Three global flexibility indexes of N-MDM2 were calculated as a function of the unfolding of the protein structure due to the increase in energy E, as shown in Figure 6. These quantities are: (i) the normalized number of inner degrees of freedom in the protein, Φ; (ii) the cluster configuration entropy, Σ; and (iii) the rigidity order parameter, Π. The index Φ is also known as the floppy mode density [23] and represents the amount of possible independent dihedral rotations in the protein chain. A rather uniform growth of Φ is observed at increasing energy (Figure 6A), indicating that the network flexibility increases almost linearly as the denaturation process takes place.

The cluster configuration entropy, Σ, is a measure of the degree of disorder in the state of the network as it descends from the classical definition of entropy in Shannon’s information theory [24] The entropy of the system is close to zero at low energy when only a single large cluster of constraints exists. By increasing the energy, a sudden increase in disorder was observed in the state of the system at 0.45 kcal mol^−1^ (Figure 6B), corresponding to a phase transition in the thermal process. A decrease was later observed because the entropy tends to reach again a null value at the end of the process since all the atoms will become independent from each other as constraints gradually disappear. From the analysis of the profile of Σ, the most relevant finding was the presence of multiple steps in this region of the curve. This observation indicates that, after the melting of the largest part of the protein structure, the unfolding of N-MDM2 proceeds in a hierarchical fashion, suggesting the presence of multiple sub-structures (i.e., intermediates).

We also monitored the rigidity order parameter Π which is the fraction of the network of constraints in the protein structure that are present at the lowest temperature and that will decrease during the denaturation process. This cluster of constraints is also named the “giant percolating cluster” because percolation theory deals with clusters of nodes in a connected network [25] and describes how adding (or removing, in this case) edges to a network leads to a phase transition consisting in a sudden dominance (or irrelevance) of this cluster for the physical properties of the system investigated. The curve (Figure 6C) showed a sharp transition at the same energy as the one observed for Σ (0.45 kcal mol^−1^), followed by a relatively lower and flatter tail in the curve. The transition point reveals the energy value at which the structure of N-MDM2 abruptly loses most of its rigidity.

In principle, the energy, E, which constitutes our reaction coordinate can be directly converted to a temperature scale [26]. Although this does not change in any way the curves observed for both Σ and Π, the interpretation of the temperature values obtained is not immediate. In fact, the onset of the phase transitions observed will depend on the protein size and fold and, in general, it may not have a direct correspondence with the unfolding temperature(s) observed in the wet-laboratory experiments. Moreover, by far the most important shortcoming is that the values obtained are highly sensitive to the specific details of the input structure used for the protein [27]. In our case, the energy of 0.45 kcal mol^−1^ observed for the phase transition of both Σ and Π would correspond to a temperature of 36 °C (a single value with no clear uncertainty associated), which can hardly be identified as a reasonable value for the actual melting point of the protein structure (when compared to the experimental values obtained by DSC and CD). To overcome this difficulty, the CNA web server offers the option to generate from a single structure an ensemble of network topologies by considering so-called “fuzzy” contributions that mimic non-covalent constraints randomly breaking or forming already in the native protein state [28]. Thus, we took advantage of this option to perform a thermal unfolding simulation of an ensemble of networks created from our single input structure, with the aim to obtain a more accurate estimate of the melting temperature of N-MDM2.

The use of an ensemble of network topologies, rather than a single one, led in our case to a surprising result: the transition of the cluster configuration entropy shifted to 1.83 ± 0.05 kcal mol^−1^ (corresponding to 63.4 ± 1.2 °C) and the transition of the rigidity order parameter to 2.16 ± 0.06 kcal mol^−1^ (70.2 ± 1.1 °C). These two values are quite different from each other, especially considering the relatively small error associated with each of them. Therefore, they correspond to two different events in the unfolding pathway of N-MDM2. The value found for Σ was still consistent with the original interpretation (see analysis above) and corresponds to the melting of the largest part of the protein structure, as observed in >90% of the conformations in the ensemble. Such temperature value (i.e., 63.4 ± 1.2 °C) is also close to the one obtained in the far-UV CD, which is especially sensitive to changes in the protein’s secondary structure. At higher temperature values, the curve of Σ still captures the denaturation of the remaining sub-structures of N-MDM2, as previously interpreted. In contrast, Π identifies a transition that we re-interpreted as corresponding to the main unfolding event along this subsequent (i.e., latest) part of the process. The temperature value obtained for the phase transition of Π (i.e., 70.2 ± 1.1 °C) is also remarkably consistent with the one observed for the highest of the two melting temperatures *T*_m_ observed with the DSC technique at 69.8 °C.

#### 2.3.2. Local Indexes in the Simulated Unfolding Process

**Figure 7:**
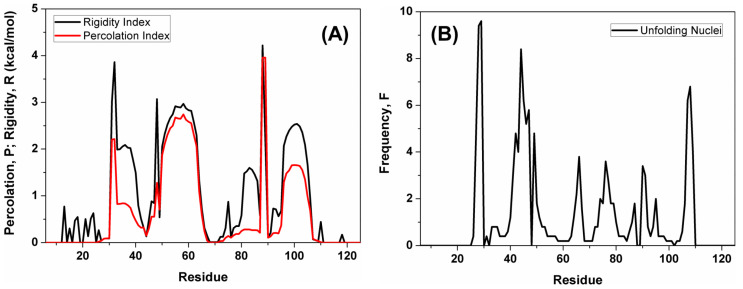
Local flexibility indexes of N-MDM2 as a function of the residue. (**A**) percolation index, *P*, and rigidity index, *R*, both related in different ways to protein rigidity. (**B**) frequency of unfolding nuclei, *F*, which is proportional to the probability of a residue to behave as a weak spot for the protein stability.

We also analyzed three local indexes that provide detailed information on the flexibility of the protein structure as a function of the residue [21]: the percolation index, *P*; the rigidity index, *R*; and the frequency of each amino acid behaving as an unfolding nucleus, *F*. All of them were calculated across the entire simulated ensemble of networks, and the results obtained are reported in Figure 7.

The percolation index, *P*, is a per-residue analogue of the rigidity order parameter Π, previously analyzed as a global index. The rigidity index *R* can be considered a generalization of *P* and represents the energy at which a bond becomes flexible, segregating from the cluster of the remaining rigid connections. Although *R* and *P* represent two distinct physical quantities, the curves obtained for N-MDM2 (Figure 7A) are largely in agreement with each other with regard to the most prominent peaks. Nevertheless, the *p* values are systematically lower than those of *R*, suggesting that the former quantity reflects unfolding events happening at a relatively lower temperature along the denaturation process. Both in the percolation and rigidity index curves, the lowest values correspond to amino acid residues possessing little or no structure already at the beginning of the unfolding process; for instance, the two protein termini. In contrast, the largest values correspond to residues that are predicted to be more rigid and resistant to denaturation. The most resilient structures were the central regions of the two helices 50–63 and 96–104, as well as the two short regions 31–32 and 88–89.

In addition, we calculated the frequency *F* of finding a residue behaving as a weak spot during the thermal unfolding [19] in the simulated ensemble of the network topologies. The values of *F* (Figure 7B) are a little bit sensitive to regions that are already largely unstructured in the native structure of the protein; for instance, the first 20 and the last 15 residues of the N and C termini of N-MDM2, respectively. In contrast, the largest values are found for residues that are part of the rigid cluster in the starting protein state, and then they segregate from it to actively operate as starting nuclei during the protein denaturation. The results pinpoint a number of regions forming distinct weak spots for the unfolding of N-MDM2, such as the short sequence regions 23–24, 39–42, and 102–103. Interestingly, whereas the first of these sequence fragments does not have a secondary structure, the last two correspond to the terminal regions of two helices.

**Figure 8:**
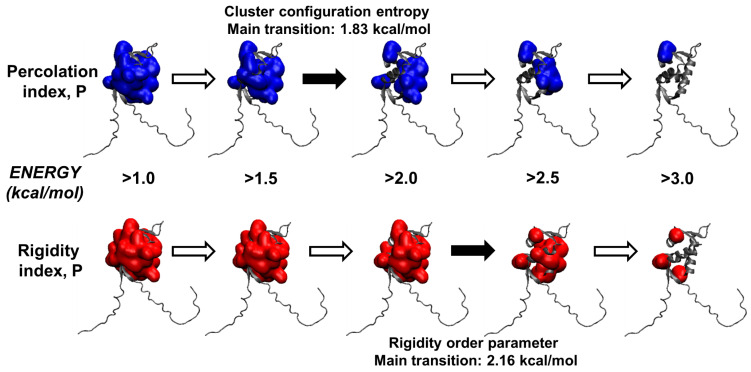
Mapping of the two local indexes *P* and *R* onto the native structure of N-MDM2 as a function of the reaction coordinate of the unfolding process. The main transitions found in the analysis of the two key global indexes, in the ensemble of networks created from the input protein structure, are indicated: the cluster configuration entropy Σ (transition at 1.83 kcal mol^−1^) and the rigidity order parameter Π (2.16 kcal mol^−1^).

Finally, the two local indexes *P* and *R* were mapped onto the three-dimensional (native) structure of N-MDM2, as shown in Figure 8, as a function of the reaction coordinate along the denaturation process (i.e., the energy E). The percolation index, *P*, which was found to be more sensitive to the early unfolding events (Figure 8), showed a distinct transition between 1.5 and 2.0 kcal mol^−1^ when the giant network cluster separates into three distinct and independent regions. Since *P* is indicative of the hierarchical organization of the network cluster, this is consistent with the main structural event previously identified by the phase transition of the cluster configuration entropy, Σ, at 1.83 kcal mol^−1^. In contrast, *R* is systematically greater than *P* and shows little differences between 1.0 and 2.0 kcal mol^−1^ (Figure 7A), whereas the most evident change takes place between 2.0 and 2.5 kcal mol^−1^. This also appears to agree with the phase transition observed in Π, the other global flexibility index previously analyzed (Section 2.3.1), with a midpoint at 2.16 kcal mol^−1^.

We note that one of the reasons for mapping *P* and *R* on the native structure of N-MDM2 is due to the practical necessity of a clear visual representation for comparing small differences in the different simulation snapshots that would otherwise be difficult to notice (Figure 8). More importantly, the main reason is due to one of the biggest limitations of the CNA, which is the fact that the analysis is restricted to the static structure of a biomolecule [21]. In fact, although CNA gives clear indications on the propensity (in terms of flexibility/rigidity) of different protein regions to move during the unfolding, it cannot predict the extent of the actual movements that will take place [29]. As an example, in our case it is not possible to assess whether the protein sub-units in the later steps of the denaturation process will tend to drift apart from each other or to remain collapsed in a molten-globule-like conformation.

To conclude, the local indexes here analyzed are in overall agreement with the global indexes formerly examined, and together they all contribute to outlining a unified picture for the main events during the unfolding of N-MDM2. Our findings point to the sheer complexity of the unfolding process, which is rather unexpected for such a small protein. Thus, they contribute to explaining the differences found in the melting temperatures in our experimental results (DSC and far-UV CD), carried out with different techniques. More importantly, this complexity is directly related to the multifaceted flexibility of the structure of N-MDM2, which we suggest should have a major role in the structural plasticity and functional versatility of this protein in the interaction with other molecular partners [30].

## 3. Discussion

It has been shown, based on NMR measurements, that N-MDM2 is a functional domain that is very sensitive to conformational rearrangements, and even at 15 °C, an evident plasticity of this protein has been observed [12] in some regions, which undergoes conformational re-adjustments to allow for the allocation of p53 [11]. These observations seem to suggest a high inner mobility of the domain and a low stability. We have tried to address these questions in our study by investigating the structural features and the conformational stability of the domain from different in vitro and in silico approaches.

The first conclusion of our work is that N-MDM2 in solution had a rather low stability (with an apparent thermal denaturation midpoint of ~55 °C) in a narrow pH interval (between pH 7.0 and 10.0). A similar low stability has been reported in other prevalently α-helical proteins [31,32,33,34], where it has been associated with a large flexibility. It is interesting to compare our results with those obtained with model proteins such as chicken and equine lysozyme [35,36]. In fact, when the specific enthalpy of denaturation in the two transitions is combined and normalized with respect to the molecular mass MW (i.e., the quantity (ΔH_m1_ + ΔH_m2_)/MW is considered), the value obtained for N-MDM2 (9.4 cal g^–1^) may seem comparable to that of chicken lysozyme (7.6 cal g^–1^) [35], which is considered a standard for the packing density of links in the native state. Nevertheless, the stability of N-MDM2 is essentially dictated by the first transition because it represents the loss of structural integrity and, very likely, will affect some of its interactions with other biomolecules. Such transition is characterized by an unfolding temperature of 52 °C and an unfolding enthalpy of 55 kcal mol^–1^, and these parameters are much lower than those for chicken lysozyme, which shows a single transition characterized by an unfolding temperature of 75 °C and an unfolding enthalpy of about 100 kcal mol^–1^. Thus, the unfolding features of N-MDM2 and chicken lysozyme are quite different: the unfolding of N-MDM2 occurs along two transitions at moderate-to-low temperatures, whereas in the case of chicken lysozyme, the unfolding takes place as a single transition at high temperatures. Furthermore, the stabilization energy is much lower for N-MDM2: at 52 °C, N-MDM2 has already lost a significant part of its structure whereas, at the same temperature, the structure of chicken lysozyme is little affected. In contrast to chicken lysozyme, when compared to N-MDM2, equine lysozyme shows a similar stability profile [36], including two transitions with low unfolding temperatures and unfolding enthalpies. We suggest that the low stability of N-MDM2 would ease the interactions with other macromolecules and it is an essential feature of this domain to carry out its function(s). 

The second conclusion of our work is that the loss of the secondary structure and the burial of solvent-exposed hydrophobic patches (as monitored by far-UV CD and ANS fluorescence), as the pH was varied, occurred in an identical manner (Figure 2). On the other hand, the acquisition of tertiary structures (as monitored by intrinsic fluorescence, Figure 2A) and compactness of the polypeptide chain (as shown by SEC, Figure 3A) occurred at a different step, although in the latter technique we cannot rule out the possibility of protein–column interactions of the partially folded structures present at acidic pH values. At acidic pH, because of the similarity of the p*K*_a_ values with those found in model peptides [37,38,39], the loss of native-like structures could be associated with the presence of aspartic or glutamic acid residues. The fact that the populated species at low pH showed a large ANS fluorescence intensity suggests that they are probably molten globules [20], as we could further confirm by the absence of thermal unfolding sigmoidal curves followed by fluorescence or CD at low pH values (Figures S2 and S4). The narrow pH range (from 7.0 to 10.0) where N-MDM2 acquired a fully native-like conformation ensures that the domain is only functional under physiological conditions.

Furthermore, we also explored the unfolding of N-MDM2. As a preliminary remark, we cannot exclude at this stage that folding of the domain, and even its stability, might be partly modified due to its isolation from the whole MDM2. In particular, we cannot rule out that interactions with other domains of the intact protein could modulate its conformational and folding features. The chemical denaturation curves with different defined steps, depending on the probe used (intrinsic fluorescence, ANS fluorescence, SEC, or far-UV CD) and the broadening of some of the transitions (e.g., that from fluorescence with large *m*-values, Figure 5) that demonstrate a low cooperativity, all contribute to indicating that the unfolding of N-MDM2 was not a two-state process [40]. These results were further supported by our SEC findings (Figure 3B).

The simulation results confirmed the structural plasticity of N-MDM2, which was especially visible in the complex nature of its unfolding process. During this reaction, the increase in flexibility of the constraint network in the protein was visible in all the global indexes analyzed (Figure 6). Moreover, the configuration entropy Σ clearly suggests a hierarchy in the melting of the different protein sub-units, which could be expected for larger proteins with a more complex architecture [21] but is more unusual for a relatively small protein such as N-MDM2. The phase transition observed for Σ corresponds to the first and main event in the denaturation process of the protein, in which the melting of the structure leads to distinct and independent sub-units. In contrast, the rigidity order parameter Π appeared to be more sensitive to later events along the unfolding pathway of N-MDM2. Local indexes in the simulated unfolding process, obtained as a function of the residue number (Figure 7) and mapped onto the native structure of N-MDM2 (Figure 8), provided specific details on the different rigidity of the various regions of this domain. The variety observed in the inner flexibility of N-MDM2 determines the presence of multiple intermediates along the unfolding process, which could also be predicted by other less-sensitive computational tools (e.g., the folding kinetic prediction tool K-Fold [41]) and, more importantly, whose presence was directly captured in our wet-laboratory experiments. The collective behavior of the sub-structures of the N-MDM2 domain, visible in the denaturing process, could be expected to be present, at least in part, also at physiological temperatures and to contribute to dictating its overall functional properties.

## 4. Materials and Methods

### 4.1. Materials

Imidazole, Trizma base, DNase, SIGMAFAST protease tablets, NaCl, Ni^2+^-resin, and Amicon centrifugal devices, with a molecular weight cut-off of 3 kDa, were from Sigma (Madrid, Spain). The β-mercaptoethanol was from BioRad (Madrid, Spain). Ampicillin and isopropyl-β-D-1-thiogalactopyranoside were obtained from Apollo Scientific (Stockport, UK). Triton X-100, Tris(2-carboxyethyl)phosphine (TCEP), dialysis tubing with a molecular weight cut-off of 3500 Da, and the SDS protein marker (PAGEmark Tricolor) were from VWR (Barcelona, Spain). The rest of the used materials were of analytical grade. Water was deionized and purified on a Millipore system. 

### 4.2. Protein Expression and Purification 

Monomeric His-tagged N-MDM2 (residues 6–125 of the intact protein) was purified as previously described [17]. The first 5 residues of N-MDM2 were missing due to optimization of the cloning sequence. Protein concentration was determined by UV absorbance, employing an extinction coefficient at 280 nm estimated from the number of tyrosines (the construct of His-tagged N-MDM2 has eight tyrosines) in the sequence [42].

### 4.3. Fluorescence

Fluorescence spectra were collected on a Cary Varian spectrofluorometer (Agilent, Santa Clara, CA, USA) interfaced with a Peltier unit. Following the standard protocols used in our laboratories, the samples were prepared the day before and left overnight at 5 °C; before experiments, samples were left for 1 h at 25 °C. Slit widths for the excitation and emission lights were 5 nm in all cases. A 1 cm-pathlength quartz cell (Hellma, Kruibeke, Belgium) was used. Concentration of N-MDM2 in the samples was 20 µM. 

#### 4.3.1. Intrinsic Fluorescence 

Protein samples were excited at 280 nm in the pH range 2.0‒12.0. The other experimental parameters have been described elsewhere [18]. Appropriate blank corrections were made in all spectra. Fluorescence experiments (pH or chemical denaturations) were repeated in triplicate with newly prepared samples. Variations of results among the experiments were lower than 10%.

The pH of each sample was measured after completion of pH denaturations with an ultra-thin Aldrich electrode in a Radiometer (Copenhagen) pH meter. The salts and acids used in the buffers have been described elsewhere [34]. The wavelength-averaged emission intensity (also called the spectrum mass center), <λ>, was calculated as described [34]. Briefly, the wavelength-averaged emission intensity, <λ>, is $<\lambda >=\sum_{1}^{n}\left(I_{i}/\lambda_{i}\right)/\sum_{1}^{n}I_{i}$, where *I*_i_ is the intensity at wavelength λ_i_. From its definition, this parameter is an integral of the value of the fluorescence spectrum, and thus, it allows one to obtain overall information on the intensities acquired in the spectrum (instead of using a single wavelength to monitor the fluorescence intensity). It is important to indicate that, conversely to the measurements of a single intensity value at a particular wavelength, <λ> is an intensive variable [43]. We have reported the value of <λ> in units of µm^−1^.

Urea denaturations at pH 7.0 (50 mM, sodium phosphate buffer), either followed by fluorescence or CD, were carried out by dilution of the proper amount of an 8 M urea stock solution. Fluorescence and CD experiments were also acquired in GdmCl (from a stock solution at concentration of 7 M). The denaturant concentration of the stock solutions was determined as described by using the refraction index of the solution [34]. All the urea or GdmCl denaturations were shown to be irreversible by following <λ>, and therefore we cannot estimate the stability in terms of the Gibbs energy, ΔG, in the denaturation of N-MDM2. This parameter, as other thermodynamic features, should have been obtained from the curves shown in Figure 5, where the variation in a spectroscopic property (raw ellipticity at a particular wavelength in CD technique; or fluorescence intensity at a particular wavelength or <λ> in fluorescence technique) is represented vs. the concentration of denaturant used.

#### 4.3.2. Thermal Denaturations

Experiments were performed at constant heating rates of 60 °C/h with an average acquisition time of 1 s and collecting points every 0.2 °C. The “average time” is the “sampling time” of the instrument at each temperature. Ideally, in a thermal scan experiment, this time should be much lower than the scan rate of the experiment to ensure that the temperature is constant during the acquisition of fluorescence emissions at a particular temperature (in our conditions, the temperature change during the sampling time was 0.017 °C, and it could be considered negligible). Thermal scans were collected at 308 nm by excitation at 280 nm from 25 to 85 °C. The rest of the experimental set was the same as described above. Thermal denaturations were not reversible at any pH value.

#### 4.3.3. ANS Binding 

The excitation wavelength was 380 nm, and emission was measured from 400 to 600 nm. Slit widths were 5 nm for excitation and emission lights. ANS stock solutions were prepared in water and diluted to yield a final concentration of 100 µM. Blank solutions were subtracted from the corresponding spectra. Protein concentration, as in the intrinsic fluorescence experiments, was 20 µM for pH and GdmCl denaturations.

#### 4.3.4. Thermal Unfolding by Using an External Probe (SYPRO Orange)

The thermal stability of N-MDM2 was assessed by differential scanning fluorimetry in a Mx3005P real-time qPCR (Agilent, Madrid, Spain). The fluorescence emission intensity of the extrinsic fluorophore SYPRO Orange (Thermo Fisher Scientific, Barcelona, Spain) was monitored as a function of the temperature at a scanning rate of 1 °C/min, using excitation and emission filters centered at 496 and 610 nm, respectively (the two available filters that were closest to the theoretical values estimated for the fluorophore excitation and emission, 491 and 586 nm). Assays were carried out at protein concentrations of 14 and 25 μM. Upon protein temperature unfolding, SYPRO Orange binds to solvent-exposed hydrophobic regions in the protein, undergoing a fluorescence quantum yield enhancement with an increase in the fluorescence intensity. The unfolding traces were analyzed according to a two-state (single-transition) unfolding model, from which the unfolding temperature and unfolding enthalpy could be estimated. Very briefly, the fluorescence intensity measured at any temperature, <*F*>, is given by

(1) $$\langle F\rangle \left(T\right)=P_{N}\left(T\right)F_{N}\left(T\right)+P_{U}\left(T\right)F_{U}\left(T\right),$$

where *P_N_* and *P_U_* are the populations of the native (N) and unfolded (U) states, and *F_N_* and *F_U_* are the intrinsic characteristic fluorescence signals for the native and unfolded states that are linear functions of the temperature:

(2) $$\begin{array}{c} F_{N}\left(T\right)=a_{N}+b_{N}T\;\\ F_{U}\left(T\right)=a_{U}+b_{U}T. \end{array}$$

The following standard relationships are considered to calculate <*F*>:

(3) $$\begin{array}{c} \begin{array}{c} ∆H\left(T\right)=∆H\left(T_{m}\right)+∆C_{P}\left(T-T_{m}\right)\\ ∆G\left(T\right)=∆H\left(T_{m}\right)\left(1-\frac{T}{T_{m}}\right)+∆C_{P}\left(T-T_{m}-T\ln\frac{T}{T_{m}}\right) \end{array}\\ \begin{array}{c} K\left(T\right)=exp\left(-∆G\left(T\right)/RT\right)\\ \begin{array}{c} P_{N}\left(T\right)=\frac{1}{1+K\left(T\right)}\\ P_{U}\left(T\right)=\frac{K\left(T\right)}{1+K\left(T\right)}, \end{array} \end{array} \end{array}$$

where *T*_m_ is the unfolding temperature, Δ*H* and Δ*C*_P_ are the differences in enthalpy and heat capacity between the unfolded and the native states, *K* is the conformational equilibrium constant, and Δ*G* is the Gibbs energy associated with the protein stability. Fitting of Equations (1) to (3) was carried out by using user-defined routines in Origin 7.0 in order to estimate *T*_m_, Δ*H*(*T*_m_), and Δ*C*_P_.

### 4.4. Circular Dichroism (CD)

Circular dichroism spectra were collected on a Jasco J810 (Tokyo, Japan) spectropolarimeter fitted with a thermostated cell holder and interfaced with a Peltier unit. The instrument was periodically calibrated with (+)-10-camphorsulphonic acid. 

#### 4.4.1. Far-UV Spectra 

Isothermal wavelength spectrum of N-MDM2 at different pH values or urea or GdmCl concentrations were acquired at a scan speed of 50 nm/min with a response time of 4 s and averaged over six scans at 25 °C in a 0.1 cm-pathlength cell. Step resolution was 0.2 nm and the bandwidth was 1 nm. Protein and buffer concentrations were the same used in intrinsic fluorescence experiments. Spectra were corrected by subtracting the baseline in all cases. The chemical and pH denaturations were repeated at least three times with new samples. The samples were prepared the day before and left overnight at 5 °C to allow for equilibration. 

#### 4.4.2. Thermal Denaturation Experiments 

The experiments were performed at constant heating rates of 60 °C/h and a response time of 8 s. Thermal scans were collected in the far-UV region by following the changes in ellipticity at 222 nm from 25 to 80 °C in 0.1 cm pathlength cells, with a total protein concentration of 20 µM. Data were collected every 0.2 °C. Solution conditions were the same as those reported in the steady-state experiments. No difference was observed between the scans aimed to test a possible drift of the signal of the spectropolarimeter. Thermal-denaturations were not reversible at any pH for N-MDM2, as shown by (i) the comparison of spectra before and after the heating; and (ii) the changes in the voltage of the instrument [44].

### 4.5. Size Exclusion Chromatography (SEC) 

This technique was used to determine the compactness of N-MDM2 [45,46], at different pH values and GdmCl concentrations, by using a ~20 µM protein concentration. Samples were loaded in 50 mM of the corresponding buffer with 150 mM NaCl (to avoid interactions with the column) and 2 mM EDTA (to avoid polypeptide degradation) in a calibrated analytical Superdex 75 10/30 HR FPLC column (GE Healthcare, Barcelona, Spain) connected to an AKTA-FPLC (GE Healthcare) at 20 °C. For GdmCl denaturation experiments, the buffer was 50 mM (Tris, pH 8.0) with 150 mM NaCl and the corresponding concentration of GdmCl. The exclusion molecular weight of the column was 70 kDa for a globular protein, according to the manufacturer and our own measurements with blue dextran. The elution volumes, *V*_e_, of N-MDM2 under different pH values were obtained from chromatogram analyses with the UNICORN software version 5.01 (GE Healthcare) from three independent measurements with freshly prepared samples. Samples of N-MDM2 previously dissolved in the corresponding running buffer were eluted at 1 mL/min and continuously monitored with an on-line detector at a wavelength of 280 nm. The samples were loaded from ice, maintaining the minimum delay possible before the injection. The standards used for column calibration, at 20 µM of protein concentration, were ribonuclease A, chymotrypsinogen A, ovalbumin, and bovine serum albumin. The *V*_e_ values of the standard proteins were measured at pH 7.0 (phosphate buffer, 50 mM) with 150 mM NaCl.

### 4.6. Differential Scanning Calorimetry (DSC)

The thermal stability of N-MDM2 was also assessed by differential scanning calorimetry in an Auto-PEAQ-DSC instrument (MicroCal, Malvern-Panalytical). The partial heat capacity of the protein solution was measured as a function of the temperature at a scanning rate of 1 °C/min under a pressure of 3.4 bars (50 psi) to avoid bubble formation and evaporation at high temperatures. Assays at protein concentrations of 14 and 25 μM were carried out, which correspond to 0.21 and 0.37 mg/mL, respectively. Several buffer-buffer scans were performed to ensure proper instrument and sample equilibration. After instrumental buffer baseline subtraction and concentration normalization, a baseline calculated from the progress of the unfolding process was subtracted to obtain the excess molar heat capacity of the protein. Because the unfolding traces showed two clear transitions, they were analyzed according to a two-transition unfolding model (i.e., considering two energetic domains), from which the unfolding temperature and enthalpy for each transition could be estimated. Very briefly, the partition function for a protein exhibiting two thermal unfolding transitions is given by

(4) $$Q\left(T\right)=1+K_{1}\left(T\right)+K_{2}\left(T\right)+K_{1}\left(T\right)K_{2}\left(T\right),$$

where *K*_i_(*T*) is the conformational equilibrium constant for each of the two domains, and the average enthalpy, <Δ*H*>, can be calculated:

(5) $$\langle ∆H\rangle \left(T\right)=RT^{2}{\left(\frac{\partial \ln Q\left(T\right)}{\partial T}\right)}_{P}=P_{1}\left(T\right)∆H_{1}\left(T\right)+P_{2}\left(T\right)∆H_{2}\left(T\right)+P_{12}\left(T\right)\left(∆H_{1}\left(T\right)+∆H_{2}\left(T\right)\right),$$

where *P*_i_ is the population of the corresponding conformational state (*P*_1_: intermediate 1; *P*_2_: intermediate 2; *P*_12_: unfolded), and Δ*H*_i_ is the difference in enthalpy between each conformational state and the native state (i.e., native state taken as a reference). The following standard relationships are considered to calculate <Δ*H*>:

(6) $$\begin{array}{c} \begin{array}{c} ∆H_{i}\left(T\right)=∆H_{i}\left(T_{m,i}\right)+∆C_{p,i}\left(T-T_{m,i}\right)\\ ∆G_{i}\left(T\right)=∆H_{i}\left(T_{m,i}\right)\left(1-\frac{T}{T_{m,i}}\right)+∆C_{p,i,i}\left(T-T_{m,i}-T\ln\frac{T}{T_{m,i}}\right) \end{array}\\ \begin{array}{c} K_{i}\left(T\right)=exp\left(-∆G_{i}\left(T\right)/RT\right)\\ P_{1}\left(T\right)=\frac{K_{1}\left(T\right)}{Q\left(T\right)},\;P_{2}\left(T\right)=\frac{K_{2}\left(T\right)}{Q\left(T\right)},\;P_{12}\left(T\right)=\frac{K_{1}K_{2}\left(T\right)}{Q\left(T\right)}, \end{array} \end{array}$$

and the average excess molar heat capacity, <Δ*C*_p_>(*T*), is calculated as the temperature derivative of <Δ*H*>:

(7) $$\langle ∆C_{P}\rangle \left(T\right)={\left(\frac{\partial \langle ∆H\rangle \left(T\right)}{\partial T}\right)}_{P},$$

which is the final relationship employed for analyzing the calorimetric thermograms. Fitting was carried out by using user-defined routines in Origin 7.0 to estimate *T*_m,i_, Δ*H*_i_(*T*_m,i_), and Δ*C*_P,i_. The populations of the four conformational states (native, intermediate 1, intermediate 2, and unfolded protein) were calculated by using the estimated parameters for each transition, as indicated above.

It could be thought that the model used is a three-state model, instead of a two-transition unfolding model. However, there is a marked conceptual difference between these two models: the three-state model considers the native state, the unfolded state, and an intermediate partially unfolded state, whereas the two-transition model considers two intermediate partially unfolded states. In mathematical terms, the three-state model is represented by a partition function containing three terms: 1 + K_1_ + K_1_K_2_, whereas the two-transition model is represented by a partition function containing four terms: 1 + K_1_ + K_2_ + K_1_K_2_. The two models may coincide when one of the intermediate states has a very low population, which occurs when the two unfolding temperatures (T_m1_ and T_m2_) are very far apart (i.e., K_2_ can be neglected compared to K_1_). However, both from a theoretical and operational point of view, we preferred to apply the two-transition model since it does not require any constraint on the intrinsic stability of the domains forming the protein.

### 4.7. Analysis of the pH, Thermal, and Chemical Denaturation Curves

The pH denaturations were analyzed, when possible, assuming that both protein species, protonated and deprotonated, contributed to the measured spectral properties:

(8) $$X=\frac{\left(X_{a}+X_{b}10^{n\left(pH-pK_{a}\right)}\right)}{\left(1+10^{n\left(pH-pK_{a}\right)}\right)},$$

where *X* is the spectral property being observed (either ellipticity at a particular wavelength or fluorescence intensity at a particular wavelength, or else <λ>); *X*_a_ is the intrinsic value of that property for the acidic species; *X*_b_ is the intrinsic value of that property observed at high pH values; p*K*_a_ is the apparent pH midpoint of the titrating group; and *n* is the Hill coefficient (which was close to 1 in all the curves reported in this work).

The thermal and chemical denaturation data for N-MDM2 were fitted to

(9) $$X=\frac{\left(X_{N}+X_{D}e^{\left(-∆G/RT\right)}\right)}{\left(1+e^{\left(-∆G/RT\right)}\right)},$$

where *R* is the gas constant and *T* is the temperature (in K). The *X_N_* and *X_D_* correspond to the intrinsic values of a physical property *X* of the native and denatured protein, respectively, being monitored. Both parameters showed a linear relationship with the temperature or the denaturant concentration. 

Although chemical denaturations of N-MDM2 were not reversible, in order to have a qualitative way to compare the different curves obtained by the several techniques used, chemical denaturation curves for N-MDM2 were analyzed according to the linear extrapolation model [34], in which the Gibbs energy is given by Δ*G* = *m*([*D*]_1/2_ − [*D*]), where [*D*] is the denaturant concentration, [*D*]_1/2_ is that at the midpoint of the transition, and *m* is the slope of the denaturant dependence of the Gibbs energy. 

The thermal denaturations, either followed by fluorescence (intrinsic or ANS) or CD, were irreversible as well. Nevertheless, we obtained an apparent thermal denaturation midpoint, *T*_m_, to allow for an estimate of the stability of N-MDM2 at the different pH values, where it was possible, and for a comparison with the DSC data from the change in Gibbs energy, Δ*G*, given by

(10) ∆GT=∆Hm1−TTm−∆Cp Tm−T+T lnTTm

where ∆*H*_m_ is the van’t Hoff unfolding enthalpy and ∆*C*_p_ is the heat capacity change in the folding reaction. The shape of Equation (10) does not impose restrictions on the value of the Δ*C*_p_ used in the fitting. 

Fitting by non-linear least-squares analysis to Equations (8) and (9), when it was possible, was carried out by using the KaleidaGraph (Abelbeck software, Reading, PA, USA) on a PC computer.

### 4.8. Simulated Thermal Unfolding

The thermal unfolding of N-MDM2 was simulated by using the CNA algorithm for rigidity analysis of a protein structure [19], implemented in the homonymous web server [21]. Default simulation parameters were used, except when explicitly stated otherwise. With the aim of reducing the bias in the choice of the starting protein structure, we used residues 6–125 of the conformation modeled by AlphaFold (entry Q00987) [22], which summarizes multiple structures of N-MDM2 deposited in the Protein Data Bank (PDB) [47].

The protein in the CNA representation consists of a body-and-bar network, with such bodies and bars modeling the macromolecule atoms and their constraints, respectively. Each atom has a maximum of six degrees of freedom, which decrease depending on the interactions it is involved in. Non-covalent interactions such as hydrogen bonds and hydrophobic tethers correspond to five and two bars, respectively. By progressively removing these non-bonded constraints, the denaturation process of the protein is mimicked. In particular, hydrogen bonds are discarded according to their estimated energy [19] from the weakest to the strongest. The removal proceeded at increasing energy E, with steps of 0.1 kcal mol^−1^, so that only hydrogen bonds with a strength E_HB_ more favorable than such threshold value (i.e., |E_HB_| ≥ E) are included in the network of each given protein state. Therefore, E represents the reaction coordinate of the simulated denaturation process.

We note that, in our definition, E has an opposite sign compared to the quantity given as output by the CNA algorithm, which is the cutoff energy E_cut_ (corresponding to removing hydrogen bonds with E_HB_ ≤ E_cut_). Our convention has some advantages: (i) E is always positive; (ii) it can be more intuitively associated with a corresponding temperature T as the latter has also a positive value; and (iii) the interpretation of the graphs is clearer as the unfolding reaction proceeds from the left to the right along the *x*-axis. The conversion from E to T is obtained by using the linear relationship by Radestock and Gohlke [26], again considering the opposite sign between E and E_cut_. Phase transition(s) at distinct temperature(s) can be identified while the network of constraints relaxes, going from more rigid to predominantly flexible states. This is visible in a variety of physical quantities that can be estimated, which reflect the degrees of freedom of the protein, its inner rigidity, the configuration entropy of the network, and other indexes that can be monitored both on a global and per-residue level (for details on how they are defined, we refer to [19,21,23] and references therein).

The analysis of the constraint network performed by using CNA, including the temperature at which phase transitions take place, can be very sensitive to the input structure of the protein. This can be improved by analyzing a structural ensemble provided from another simulation technique such as classical molecular dynamics (MD) [48] or, as an alternative, by producing an ensemble of networks starting from the single structure provided [28]. The CNA web server offers an option to generate an ensemble of network topologies from the input structure by considering so-called “fuzzy” contributions that mimic non-covalent constraints randomly breaking or forming already in the native protein state [28]. We exploited this option and performed our analysis on 500 network topologies, which is ten times larger than the number suggested as default.

## 5. Conclusions

MDM2 is a protein that can sample a large variety of conformations. Among the only three well-folded domains it contains, the N-terminal region N-MDM2 is the largest and plays a key role in the interaction of the whole protein with several molecular partners. In spite of being well-folded, N-MDM2 has its own distinctive features in terms of structural flexibility and plasticity. In this work, we have studied how the flexibility of N-MDM2 reflects its structural stability and, conversely, how the unfolding process encodes significant properties on the behaviour of this domain at physiological temperatures. The results obtained provide an overall picture on the sensitivity of N-MDM2 to changes in the surrounding environment, including temperature, pH, and the presence of chemical denaturants. In particular, N-MDM2 shows a very low stability and a hierarchical structure in the denaturation process that suggests a delicate organization of its sub-structures with the presence of various intermediates. 

The overall findings suggest that a large conformational flexibility is an intrinsic feature of the N-terminal region of MDM2. Therefore, it is immediate to speculate that these properties are crucial for the function of N-MDM2 and especially for the interaction with p53 and the other proteins belonging to the same regulatory network. In addition to providing insights on an such isolated domain, our studies may set the basis to exploit this knowledge in some applications—as an example, to find new inhibitors of the interaction between p53 and N-MDM2 by using thermal stability measurements. More generally, the properties of N-MDM2 can also be fundamental for the interaction with additional molecular partners unrelated to p53, many of which have been uncovered in recent years.

## Data Availability

The datasets generated and/or analyzed during the current study are available from the corresponding authors on reasonable request.

## Supplementary Materials

The following supporting information can be downloaded at: https://www.mdpi.com/article/10.3390/molecules28227578/s1, Figure S1: changes upon pH variations in intrinsic and ANS fluorescence title; Figure S2: thermal denaturations followed by the intrinsic fluorescence at selected pH values; Figure S3: far-UV CD spectra at two selected pH values; Figure S4: thermal denaturations followed by far-UV CD; Figure S5: raw data of the DSC thermogram before baseline subtraction; Figure S6: far-UV CD (raw ellipticity at 222 nm).

## Abbreviations

ANS	8-anilinonapthalene-1-sulfonic acid
CD	circular dichroism
CNA	constraint network analysis
DSC	differential scanning calorimetry
GdmCl	guanidinium hydrochloride
IDP	intrinsically disordered protein
MDM2	murine double minute 2 oncoprotein
N-MDM2	N-terminal region of MDM2 (residues 6‒125 of the intact protein)
PDB	Protein Data Bank
SEC	size exclusion chromatography
TA	transactivation domain
UV	ultraviolet
