# Supplementary material for: Conformational Stability of the N-Terminal Region of MDM2
Source: Molecules. 2023 Nov 14;28(22):7578. doi: 10.3390/molecules28227578 (PMC10673428; doi:10.3390/molecules28227578)
Supplement: Supplementary file 1 [file molecules-28-07578-s001.zip › molecules-2682833-supplementary.pdf]

Supplementary Information for the manuscript

Conformational stability of the N-terminal region of MDM2

Bruno Rizzuti, Olga Abian, Adrián Velázquez-Campoy and José L. Neira

SUPPLEMENTARY FIGURE LEGENDS

Figure S1: **pH-induced structural changes of N-MDM2 followed by spectroscopic techniques**: (A) Variations in the intensity at 310 after excitation at 280 nm. (B) Variations in the ANS-fluorescence of N-MDM2 monitored by the changes in the <λ>.

Figure S2: **Thermal denaturations at different pH values of N-MDM2 monitored by fluorescence**: The thermal denaturations followed by the intrinsic fluorescence at selected pH values.

Figure S3: **Far-UV CD spectra of N-MDM2**: Far-UV CD spectra of the isolated domain at two selected pH values.

Figure S4: **Thermal denaturations at different pH values of N-MDM2 monitored by far-UV CD**: The thermal denaturations followed by the changes in ellipticity at 222 nm at selected pH values.

Figure S5: **Urea denaturations of N-MDM2**: (A) Changes of the ellipticity at 222 nm (far-UV CD). (B) Changes of the intrinsic fluorescence monitored by the <λ>, after excitation at 280. (B) Changes of the ellipticity at 222 nm (far-UV CD). Experiments were carried out at 25 ºC in phosphate buffer (50 mM, pH 7.0).

Figure S6: **DSC of N-MDM2**: Raw data of DSC scan of N-MDM2 (before baseline substraction).

Figure S1

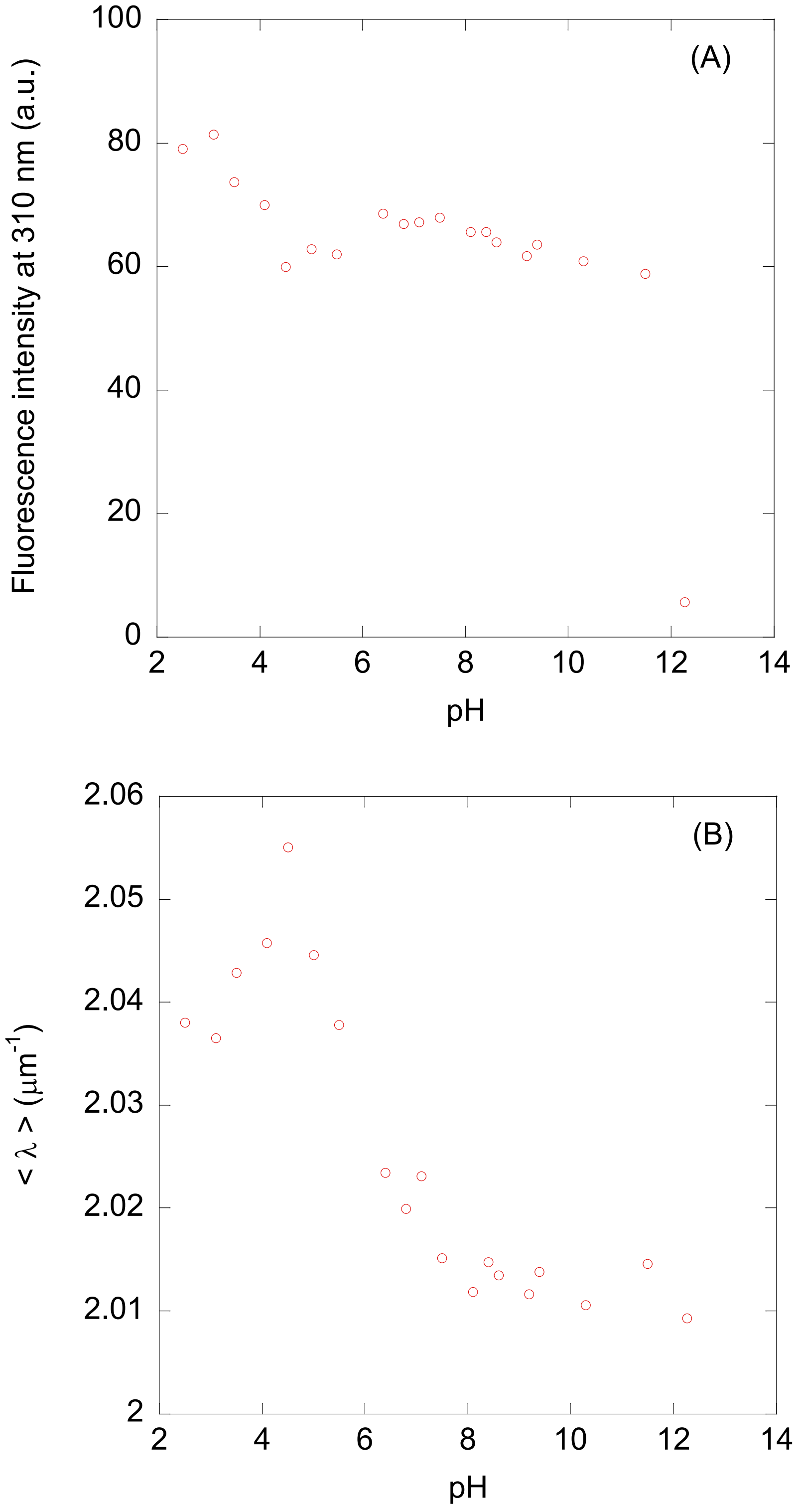

(A)
Fluorescence intensity at 310 nm (a.u.)
100
80
60
40
20
0
2
4
6
8
10
12
14
pH
(B)
< λ > (μm-1)
2.06
2.05
2.04
2.03
2.02
2.01
2
2
4
6
8
10
12
14
pH

Figure S2

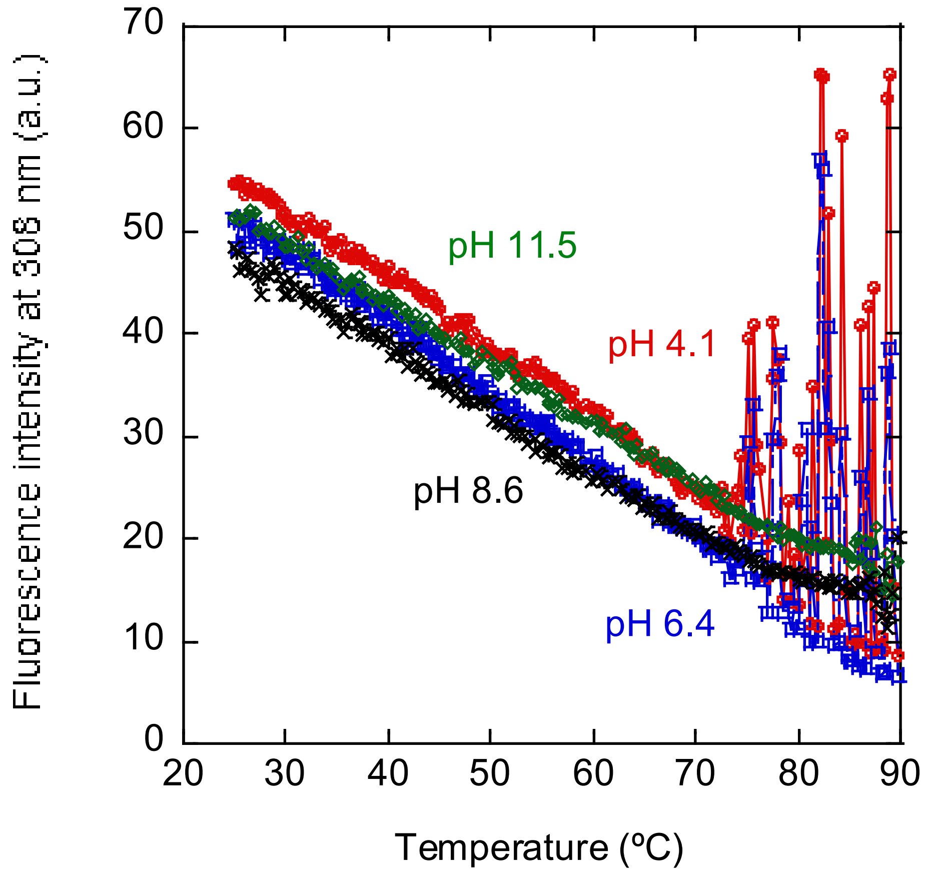

70
60
50
40
30
20
10
0
20
30
40
50
60
70
80
90
Fluorescence intensity at 308 nm (a.u.)
Temperature (ºC)
pH 11.5
pH 4.1
pH 8.6
pH 6.4

Figure S3

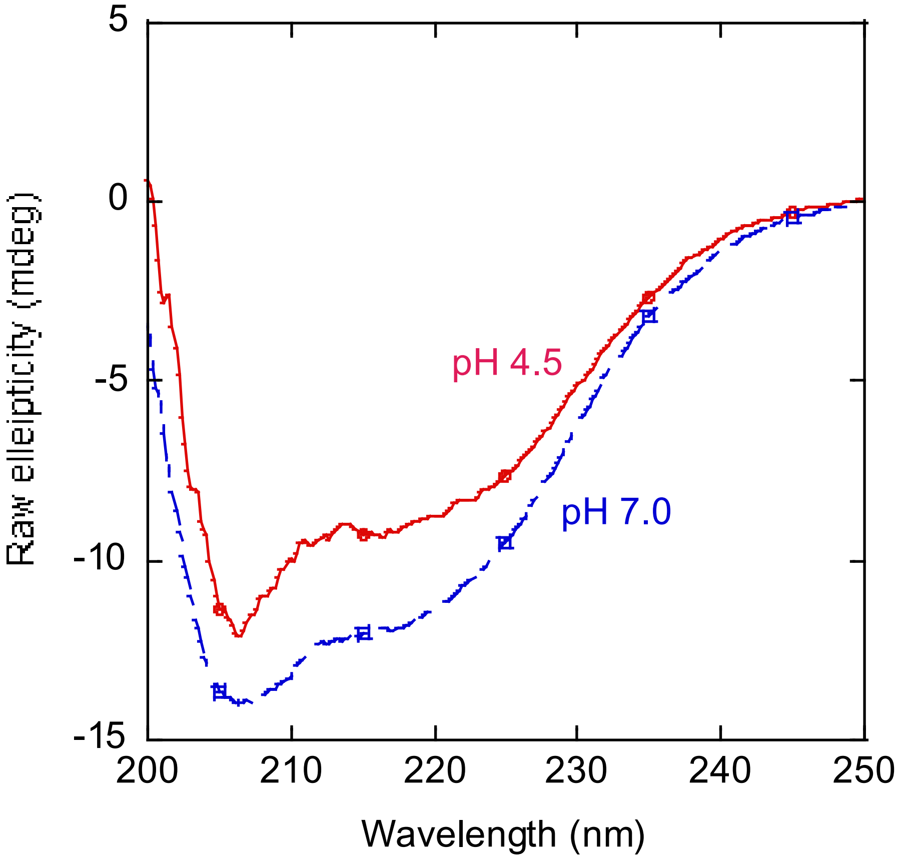

5
0
-5
-10
-15
200
210
220
230
240
250
Raw elleipticity (mdeg)
Wavelength (nm)
pH 4.5
pH 7.0

Figure S4

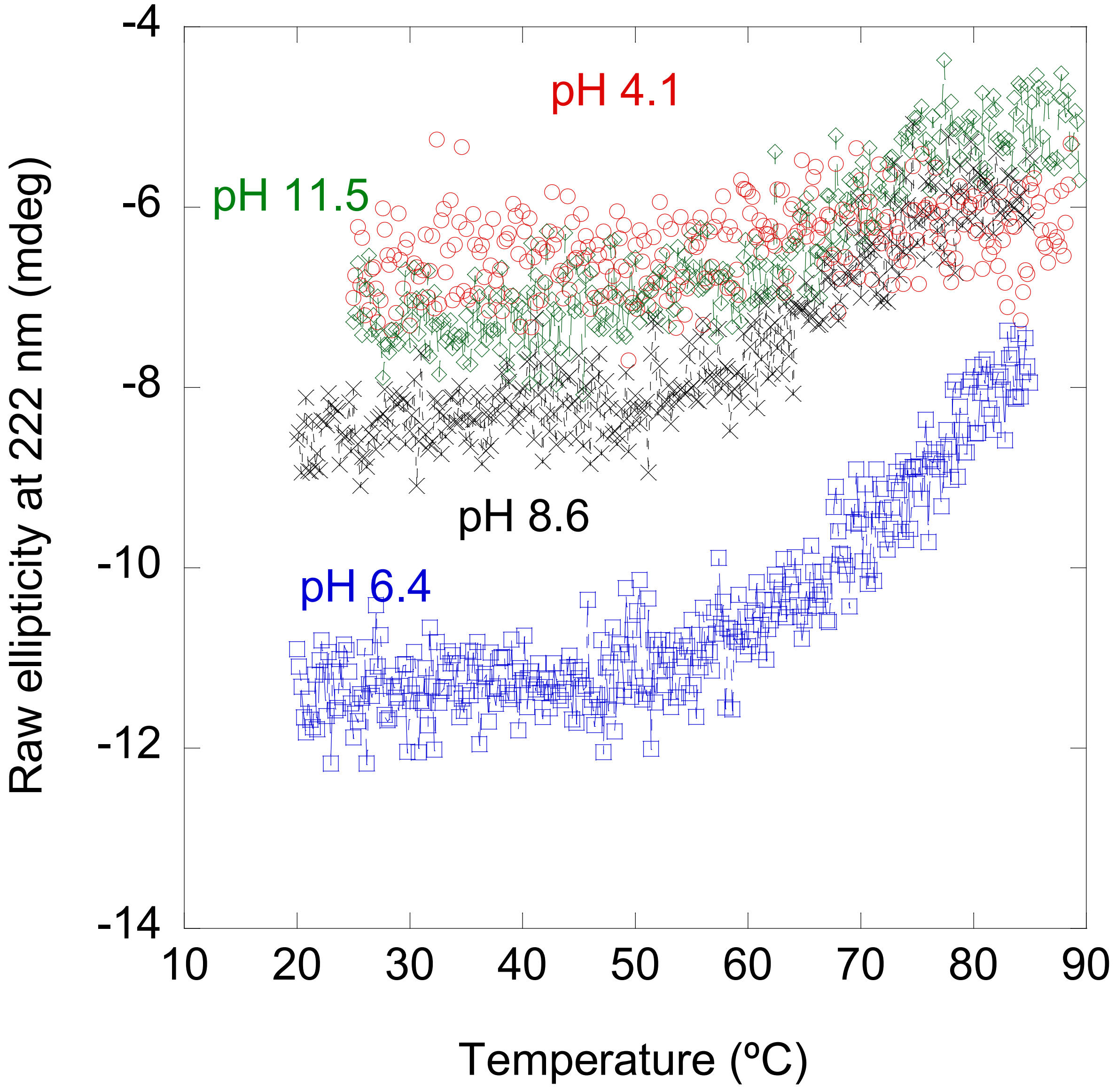

-4
-6
-8
-10
-12
-14
10
20
30
40
50
60
70
80
90
pH 4.1
pH 11.5
pH 8.6
pH 6.4
Raw ellipticity at 222 nm (mdeg)
Temperature (ºC)

Figure S5

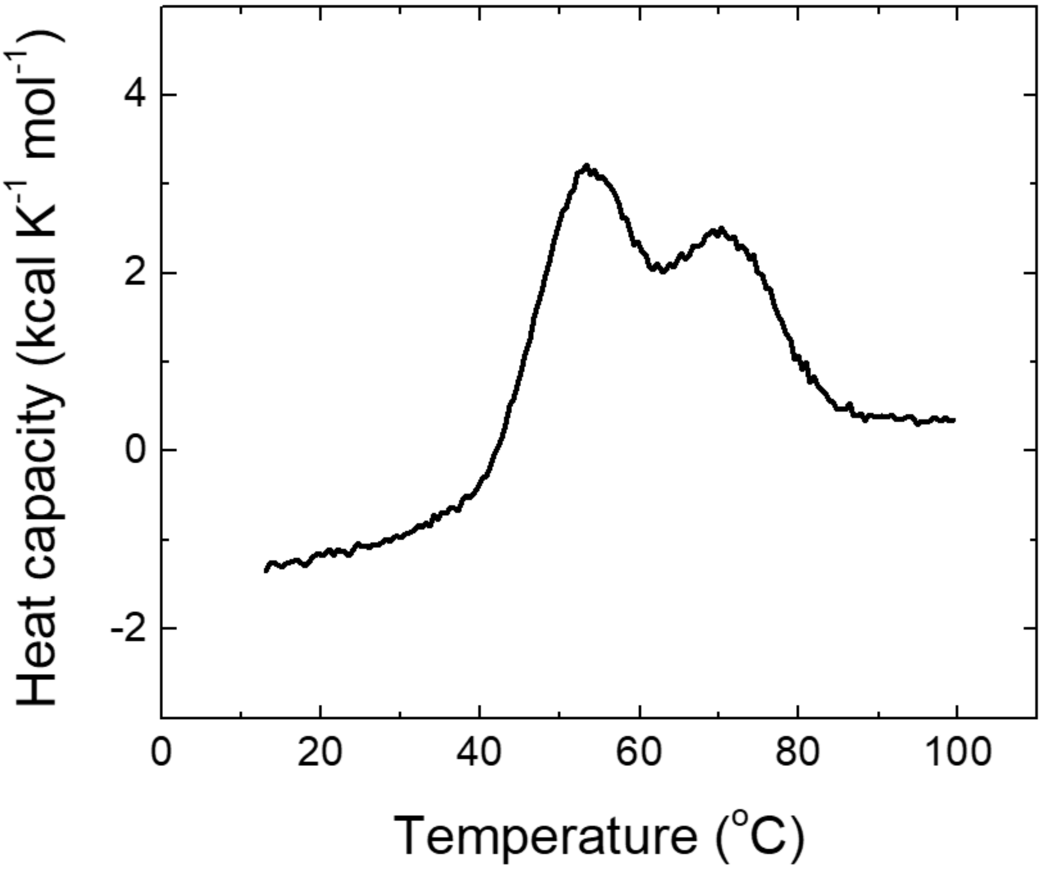
Heat capacity (kcal K^{-1} mol^{-1})
4
2
0
-2
0
20
40
60
80
100
Temperature (°C)

Figure S6

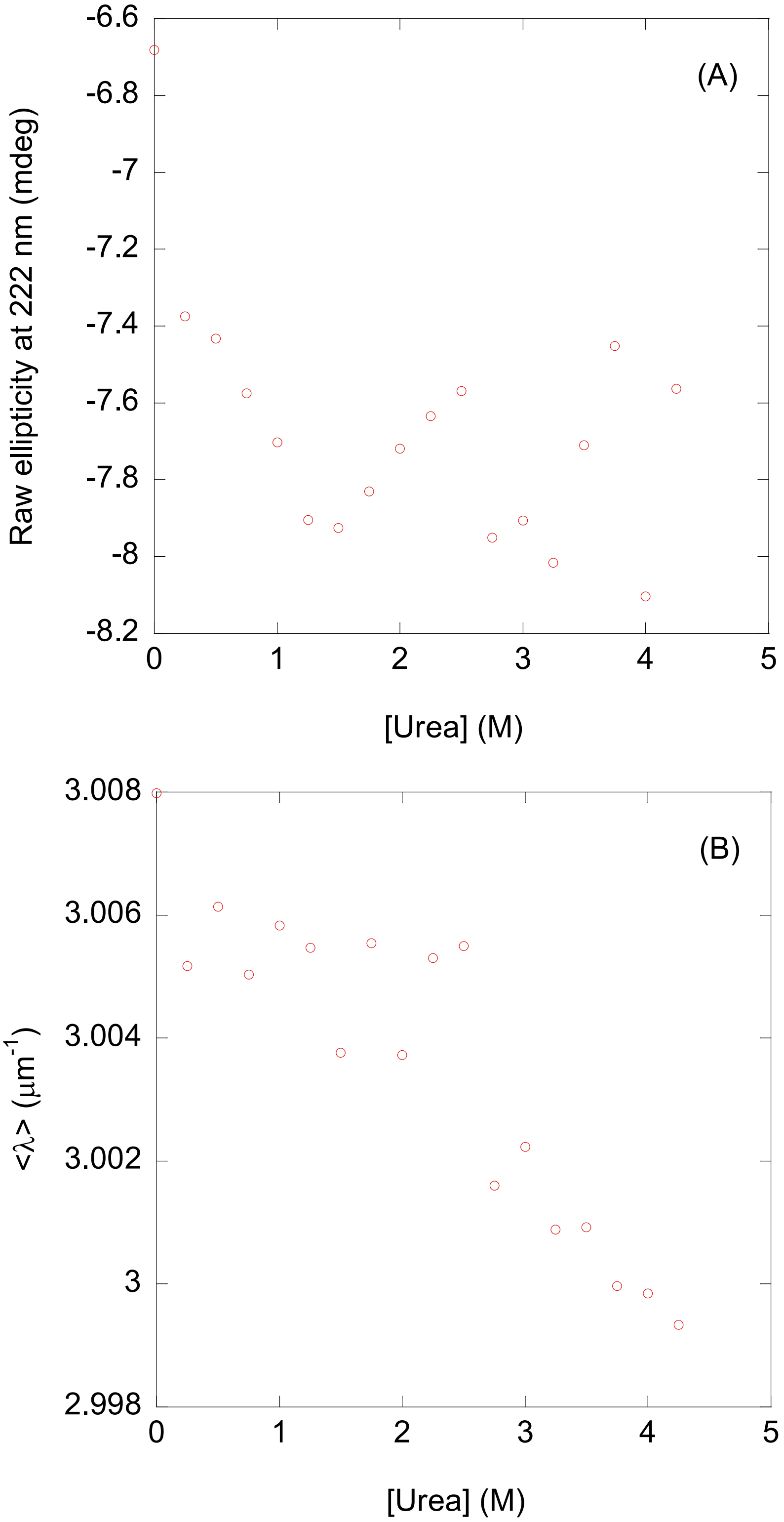
(A)
Raw ellipticity at 222 nm (mdeg)
-6.6
-6.8
-7
-7.2
-7.4
-7.6
-7.8
-8
-8.2
0
1
2
3
4
5
[Urea] (M)
(B)
<λ> (μm^{-1})
3.008
3.006
3.004
3.002
3
2.998
0
1
2
3
4
5
[Urea] (M)
